# Health-related quality of life after myocardial infarction is associated with level of left ventricular ejection fraction

**DOI:** 10.1186/1471-2261-8-28

**Published:** 2008-10-12

**Authors:** Kjell I Pettersen, Elena Kvan, Arnfinn Rollag, Knut Stavem, Aasmund Reikvam

**Affiliations:** 1 Norwegian Knowledge Centre for the Health Services, Oslo, Norway; 2 Medical Division, Akershus University Hospital, Lørenskog, Norway; 3 Department of Pharmacotherapeutics, Faculty Division Rikshospitalet, Faculty of Medicine, University of Oslo, Oslo, Norway; 4 Helse Øst Health Services Research Centre, Lørenskog, Norway; 5 Faculty Division Akershus University Hospital, Faculty of Medicine, University of Oslo, Lørenskog, Norway

## Abstract

**Background:**

The objective was to explore the relationship between left ventricular ejection fraction (LVEF) assessed during hospitalization for acute myocardial infarction (MI) and later health-related quality of life (HRQoL).

**Methods:**

We used multivariable linear regression to assess the relationship between LVEF and HRQoL in 256 MI patients who responded to the Kansas City Cardiomyopathy Questionnaire (KCCQ), the EQ-5D Index, and the EuroQol Visual Analogue Scale (EQ-VAS) 2.5 years after the index MI.

**Results:**

167 patients had normal LVEF (>50%), 56 intermediate (40%–50%), and 33 reduced (<40%). The mean (SD) KCCQ clinical summary scores were 85 (18), 75 (22), and 68 (21) (*p *<0.001) in the three groups, respectively. The corresponding EQ-5D Index scores were 0.83 (0.18), 0.72 (0.27), and 0.76 (0.14) (*p *= 0.005) and EQ-VAS scores were 72 (18), 65 (21), and 57 (20) (*p *= 0.001). In multivariable linear regression analysis age ≥ 70 years, known chronic obstructive pulmonary disease (COPD), subsequent MI, intermediate LVEF, and reduced LVEF were independent determinants for reduced KCCQ clinical summary score. Female sex, medication for angina pectoris at discharge, and intermediate LVEF were independent determinants for reduced EQ-5D Index score. Age ≥ 70 years, COPD, and reduced LVEF were associated with reduced EQ-VAS score.

**Conclusion:**

LVEF measured during hospitalization for MI was a determinant for HRQoL 2.5 years later.

## Background

Left ventricular ejection fraction (LVEF) is the single most used non-invasive measure of cardiac function in clinical practice and is an important prognostic factor for survival after myocardial infarction (MI), in stable coronary artery disease (CAD), and in heart failure [1-3]. Health-related quality of life (HRQoL) has also been identified as a predictor of survival in patients with CAD and heart failure [4-7].

However, the relationship between LVEF and HRQoL has not been settled and whether LVEF can predict HRQoL is still controversial. Some studies have observed an association [8-11] while others have not [12-17]. Most of these studies reported on highly selected heart patients enrolled in clinical trials [9,13,14], patients with known reduced LVEF [13,14], and patients with chronic heart failure [11,17]. Only two previous studies included unselected MI patients, of which one observed an association between LVEF measured at the time of the MI and later HRQoL [10,16]. Better understanding of the relationship between cardiac function and quality of life might contribute to tailor treatment that would maintain or improve the patients' daily functioning. Evidently, more research on this subject is needed.

Accordingly, our aim was to assess the relationship between LVEF measured during hospitalization for acute MI and HRQoL 2.5 years later in an unselected MI patient population, using both a generic and a disease-specific HRQoL measure.

## Methods

### Study design and sample

**Figure 1:**
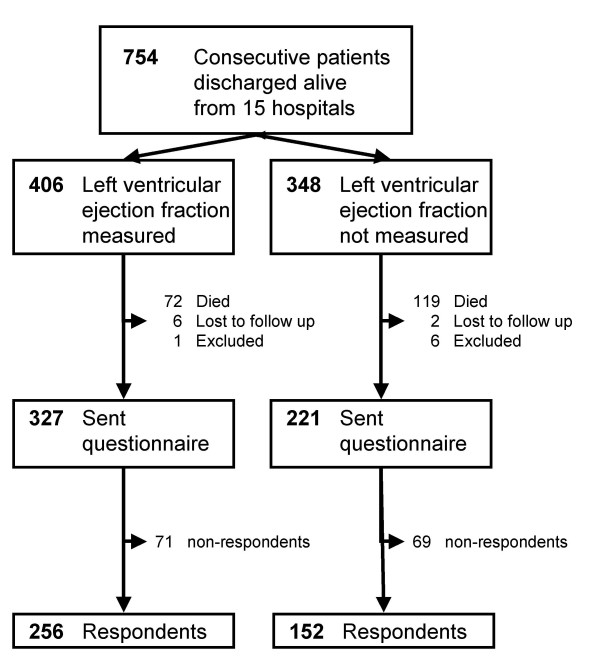
**Flow chart of study population**. Study population stratified on patients with and without their left ventricular ejection fraction measured

We established a cohort of hospitalized patients with a discharge diagnosis of acute MI, defined as codes I21 and I22 in ICD-10 (The International Statistical Classification of Diseases and Related Health Problems, tenth revision) [18]. We wanted our cohort to be representative of Norwegian patients with MI, and hence recruited them from teaching and non-teaching hospitals in different regions of Norway. We included a total of 754 consecutive patients who were discharged alive from 15 hospitals during a 3-month period between August 1, 1999, and January 31, 2000. Before discharge, LVEF was measured in 406 (54%) patients. The patient population is described in Figure 1.

In 2002, we mailed a questionnaire to patients who were still alive according to hospital information systems and the National Population Register of Statistics Norway. The questionnaires were mailed from the hospital of discharge, along with a cover letter signed by the head of the hospital's cardiology unit. After 4 weeks, we sent a reminder to non-respondents.

At the time of the survey, 191 of the 754 patients had died, 8 had an unknown address, and 7 were excluded for miscellaneous reasons. Hence, we mailed the questionnaire to the remaining 548 patients of whom 408 (74%) returned completed questionnaires. A total of 256 of the 408 respondents (63%) had data on LVEF (Figure 1). The mean time from the index MI to questionnaire response was 2.5 (SD = 0.2) years, range 2.1–3.1 years.

### Review of medical records

Baseline characteristics were abstracted from the patients' medical records and included previous medical history, presenting features, in-hospital treatment, and medication at discharge. Cardiovascular morbidities from before the index MI were categorized as previous MI, hypertension, angina pectoris, heart failure, peripheral vascular disease, or stroke. The diagnoses were based on either previous ICD-codes or explicit statements in the medical record. The major indications for each cardiovascular drug prescribed at discharge were classified as secondary prevention, hypertension, angina pectoris, heart failure, or other. More details about sampling and collection of data from the patients' medical records are available elsewhere [18].

### Measurement of left ventricular ejection fraction

We measured LVEF by one of two methods: Multiple gated-acquisition radionuclide ventriculography (MUGA) in 129 (51%) patients and echocardiography in 127 (49%) patients. MUGA is a reliable reference method, and the widely used echocardiographic method correlates fairly well with radionuclide imaging [19,20]. LVEF >50% is considered to indicate normal left ventricular function, while LVEF <40% indicates reduced function. According to this classification we categorized LVEF as normal (LVEF >50%), intermediate (40–50%) or reduced (<40%).

### Questionnaire

The questionnaire focused on HRQoL, using the EQ-5D Index, the EQ-5D Visual Analogue Scale (EQ-VAS), and the Kansas City Cardiomyopathy Questionnaire (KCCQ). In addition, we asked about subsequent cardiac events and revascularization procedures after hospital discharge for the index MI.

The KCCQ is a self-administered 23-item questionnaire designed for measuring HRQoL in patients with chronic heart failure. It comprises six scales: Symptoms, Symptom stability, Physical limitation, Social limitation, Self-efficacy and Quality of life [21]. Four of the scales, Symptoms, Physical limitation, Social limitations and Quality of life, are aggregated to an overall score, the KCCQ clinical summary score [21]. Each item is scored on a 5 to 7-point Likert scale [22]. Each scale score is calculated as the mean of its item scores and transformed to a 0–100 scale, with higher score indicating higher level of functioning. The KCCQ has been translated into Norwegian, and its psychometric properties have been documented in post MI patients [23]. Change in KCCQ score of 5, 10, and 15 points correspond with small, moderate, and large clinical change respectively [24].

The EQ-5D is a self-administered HRQoL instrument with 5 items: Mobility, Self-care, Usual activities, Pain/discomfort, and Anxiety/depression. Each item is scored on a 3-point Likert scale: no problems (score of 1), moderate problems (2), and extreme problems (3). Responses to these items can be converted to a utility score, the EQ-5D Index, by applying an algorithm derived from time trade-off valuations of health status obtained from the general population [25]. A score of 1.0 represents perfect health and 0 represents dead. For the EQ-5D Index negative utilities are possible, representing states perceived to be worse than dead. We used a UK time trade-off tariff [26]. The EQ-5D Index has been used and documented in post MI patients [27-29]. In addition to the QE-5D Index the EQ-5D questionnaire include the EQ-VAS, a visual analogue scale ranging current overall health by one single number on a scale from 0 (worst imaginable health state) to 100 (best imaginable health state) [28]. The EQ-VAS has documented acceptable reliability and validity in patients with CAD [29-31].

### Statistical analysis

We present descriptive statistics with means and SDs, or proportions. For group comparisons, we used the *t*-test, analysis of variance, Kruskal-Wallis test for three independent groups, Wilcoxon rank-sum test, or chi-square test where appropriate.

We used multiple linear regression analysis to identify determinants for KCCQ clinical summary score, EQ-5D Index, and EQ-VAS at a mean of 2.5 years after MI. To reduce problems with multicollinearity, we checked pairwise Pearson correlations (*r*) between independent variables. However, none of the pairwise correlations had *r *> 0.70. Variables with *p *< 0.25 in bivariable linear regression analysis were included in multivariable modeling. In the multivariable models, we first included age at admission, sex, length of education in years, and LVEF in the models, and then added the other potential independent determinant variables in a forward stepwise fashion. In addition to age, sex, education, and LVEF, we retained all independent variables with p < 0.05 in multivavariable linear regression in the final model. The final models were checked for interactions.

The coefficient of determination (*R*-square) is a measure of explained variance in regression analysis. To determine the marginal contribution of LVEF to *R*-square, in KCCQ clinical summary score, EQ-5D Index score, and EQ-VAS, we compared the *R*-square in the final models adjusted for degrees of freedom, with *R*-square in the final models without LVEF.

We used a 5% significance level with two-sided tests. Standard statistical software was used for all analyses (SPSS version 12.0, SPSS, Chicago, IL). The Regional Committee for Medical Research Ethics and the Norwegian Data Inspectorate approved the study.

### Demographics

Respondents (*n *= 408) were younger than non-respondents (*n *= 140), comprised a higher proportion of males, had less cardiovascular morbidity at admission, and fewer cardiovascular indications for medication at discharge. Respondents and non-respondents did not differ in the proportion of patients with ST-segment elevation at admission, Q-wave infarction, or localization of the index MI. Respondents who had their LVEF measured (*n *= 256) had higher LVEF than non-respondents who had their LVEF measured (*n *= 71) (normal, intermediate, reduced: 65%, 22%, 13% vs. 51%, 27%, 23%, *p *= 0.02). A more extensive comparison of respondents and non-respondents has been presented elsewhere [23].

## Results

Respondents who had their LVEF measured (*n *= 256) during hospitalization for the index MI were younger than respondents who had not (*n *= 152), had less cardiovascular morbidity at admission, and a higher proportion were smokers. Further, a larger proportion of respondents who had their LVEF measured had ST-segment elevation MI, underwent acute revascularisation, and developed Q-wave MI, and a lower proportion had medication for angina pectoris at discharge (Table 1).

**Table 1:** Possible determinant variables

	*All respondents*			*LVEF value*			
	
	*LVEF measured*	*LVEF not measured*	*p*	*Normal (>50%)*	*Intermediate (40 – 50%)*	*Reduced (<40%)*	*p*
*n*	256	152		167	56	33	
Time since index MI, years	2.5 (0.2)	2.6 (0.2)	<0.001	2.5 (0.2)	2.6 (0.2)	2.5 (0.2)	0.046
Sex (% women)	29	29	1.0	29	30	27	1.0
Age, years	64 (12)	68 (12)	0.002	62 (12)	68 (11)	68 (13)	0.001
Education, years, range 7–21	10 (3)	10 (4)	0.9	11 (3)	10 (3)	10 (3)	0.3
Smoker at admission	49^a^	39^b^	0.07	51^c^	48^d^	45^e^	0.8
*Comorbidities before the index MI*							
Diabetes mellitus	10	6	0.3	8	11	15	0.5
Chronic obstructive pulmonary disease	8	7	0.7	6	13	6	0.2
Hypertension	29	35	0.2	30	29	24	0.8
Previous MI	17	24	0.08	11	24	36	0.001
Angina pectoris	22	30	0.08	20	22	33	0.2
Peripheral vascular disease	4	9	0.08	2	7	9	0.1
Stroke	5	6	0.5	3	7	12	0.07
Heart failure	2	8	0.009	1	2	9	0.02
*Index MI, characteristics*							
ST-segment elevation	59	35	<0.001	58	70	46	0.07
Localization			0.1				<0.001
Anterior wall	44	30		34	57	70	
Inferior wall	41	38		51	27	12	
Unknown	16	24		15	16	18	
Q-wave	48	33	0.004	47	48	52	0.9
*In-hospital treatment*							
Revascularization	49	22	<0.001	48	63	33	0.02
*Indication for medication at discharge*							
Secondary prevention	99	98	0.7	99	100	97	0.4
Hypertension	11	12	0.8	14	5	6	0.1
Angina pectoris	18	35	<0.001	19	18	12	0.6
Heart failure	19	22	0.4	7	29	61	<0.001
*Subsequent events*							
New MI	5	7	0.5	5	5	3	0.8
Percutaneous coronary intervention	15	11	0.3	20	5	6	0.008
Coronary artery bypass graft surgery	11	13	0.7	12	7	15	0.5

Background variables, characteristics of the index myocardial infarction, treatment and subsequent events according to whether left ventricular ejection fraction (LVEF) was measured or not and in patients with normal (>50%), intermediate (40–50%), and reduced (<40%) LVEF. Numbers represent mean (SD) or percent.

^a ^*n *= 227; ^b ^*n *= 137; ^c ^*n *= 150; ^d ^*n *= 48; ^e ^*n *= 29; MI, Myocardial Infarction

Among respondents who had their LVEF measured, those with reduced LVEF were older and had increased prevalence of heart failure at admission. There was an increased prevalence of previous MI, anterior wall infarction, and use of medication on the indication heart failure with falling LVEF. Further a smaller proportion of respondents with reduced LVEF underwent acute revascularization or had subsequent percutaneous coronary intervention (Table 1).

### Health-related quality of life

The mean score for all patients with LVEF measured was 80 for the KCCQ clinical summary scale, 0.80 for the EQ-5D Index, and 69 for the EQ-VAS. HRQoL scores differed between patients with different levels of LVEF both for the KCCQ clinical summary score (*p *< 0.001) the EQ-5D Index (*p *= 0.005), and the EQ-VAS (*p *= 0.001) (Table 2).

**Table 2:** Health-related quality of life scores

	*Left ventricular ejection fraction*		
	
	*Normal (>50%)*	*Intermediate (40–50%)*	*Reduced (<40%)*
*n*	167	54	33
KCCQ clinical summary score	85 (18)	75 (22)	68 (21)
			
*n*	160	53	30
EQ-5D Index	0.83 (0.18)	0.72 (0.27)	0.76 (0.14)
			
*n*	148	46	26
EQ-VAS	72 (18)	65 (21)	57 (20)

KCCQ clinical summary score, EQ-5D Index score, and EQ-VAS score according to left ventricular ejection fraction, mean (SD)

KCCQ, Kansas City Cardiomyopathy Questionnaire; EQ-VAS, EuroQol Visual Analogue Scale

In multivariable linear regression analysis, age ≥ 70 years, a history of chronic obstructive pulmonary disease (COPD), subsequent MI, and intermediate or reduced LVEF measured during hospitalization were all independent determinants of lower KCCQ clinical summary score 2.5 years after the index MI (Table 3). Female sex, medication for angina pectoris at discharge, and intermediate LVEF were independent determinants a lower EQ-5D Index score, while a history of peripheral vascular disease was associated with a higher EQ-5D Index score (Table 3). Age 70 ≥ years, a history of COPD, and reduced LVEF were independent determinants of lower EQ-VAS score (Table 3).

**Table 3:** Multivariable linear regression analyses

	*KCCQ clinical summary score*			*EQ-5D Index*			*EQ-VAS*		
	
	*B*	*(95%CI)*	*p*	*B*	*(95%CI)*	*p*	*B*	*(95%CI)*	*p*
*n*	253			242			219		
Sex (0 = male, 1 = female)	-3.4	(-8.8 to 2.1)	0.2	-0.10	(-0.16 to -0.04)	0.001	-1.7	(-7.7 to 4,2)	0.6
Age < 70 years = 0, ≥ 70 years = 1	-6.9	(-12.1 to -1.6)	0.01	-0.03	(-0.09 to 0.03)	0.3	-6.4	(-12.0 to -0,7)	0.03
Education, years (range 7–21)	0.5	(-0.3 to 1.2)	0.2	-0.00	(-0.01 to 0.01)	0.5	-0.09	(-0.9 to 0.7)	0.8
Left ventricular ejection fraction									
>50%									
40–50%	-6.9	(-13.0 to -0.9)	0.02	-0.10	(-0.17 to -0.04)	0.001	-4.3	(-10.7 to 2.1)	0.2
<40%	-15.4	(-22.6 to -8.3)	<0.001	-0.07	(-0.14 to -0.01)	0.08	-13.5	(-21.4 to -5.6)	0.001
Peripheral vascular disease				0.16	(0.04 to 0.28)	0.01			
Chronic obstructive pulmonary disease	-12.5	(-21.5 to -3.5)	0.006				-12.1	(-21.5 to -2.8)	0.01
Medication for angina pectoris at discharge				-0.11	(-0.17 to -0.04)	0.001			
Subsequent myocardial infarction	-11.2	(-21.9 to -0.6)	0.04						
Total adjusted *R*-square	0.16			0.16			0.10		

Independent determinants of health-related quality of life 2.5 years after myocardial infarction in patients with left ventricular ejection fraction measured during index hospitalization.

In the final multivariable models, *R*-square was 0.16 for both KCCQ clinical summary score and EQ-5D Index, and 0.10 for the EQ-VAS (Table 3). When excluding LVEF in the final models, *R*-square was 0.09, 0.12, and 0.06 respectively, indicating that LVEF accounted for 25% to 44% of the variation explained by the final multivariable models.

One hospital measured LVEF routinely in all MI patients, using MUGA (*n *= 101). When applying our final multivariable models in this subset of patients, the relationship between LVEF and HRQoL was essentially the same as in all patients with measured LVEF. In this subset, using KCCQ clinical summary score as the dependent variable, the unstandardized regression coefficient (*B*) and 95% confidence interval (CI) for patients with intermediate LVEF and reduced LVEF were -10.4 (-21.0 to 0.3) (*p *= 0.06) and -13.6 (-26.9 to -0.4) (*p *= 0.04) respectively. With EQ-5D Index as dependent variable *B *and 95% CI were -0.13 (-0.25 to -0.02) (*p *= 0.02) and -0.02 (-0.16 to 0.12) (*p *= 0.7).

## Discussion

In this study LVEF measured during hospitalization for the index MI was a determinant of HRQoL 2.5 years later, with poorer HRQoL in patients with reduced LVEF. This relationship persisted after adjusting for comorbidities, sociodemographic variables, and variables related to the index MI. The summary score of the condition-specific KCCQ questionnaire, which has been validated in an earlier study [23], and the EQ-VAS showed a trend of falling scores with falling LVEF, while the EQ-5D Index only captured a difference between patients with normal and intermediate LVEF. A difference in score for the KCCQ clinical summary score between patients with normal LVEF and intermediate or reduced LVEF of 10 and17 points respectively, indicates a moderate to large clinical difference between the groups of patients with different LVEF [24].

The observed falling HRQoL scores with falling LVEF both for KCCQ clinical summary score and EQ-VAS indicate that the level of systolic heart function is a determinant for HRQoL. However, some influence from the mere fact that an MI has occurred cannot be ruled out since also patients with normal LVEF tend to have reduced EQ-5D scores compared to US norms [32]. If so, it is possible that other cardiac pathophysiological mechanisms or psychological mechanisms are involved. An understanding of the way LVEF may influence HRQoL can be provided from a conceptual model outlined by Wilson and Cleary [33]. In their model biological and physiological variables influence symptoms, that is, patients with reduced LVEF may experience symptoms such as fatigue, dyspnoea, and sleep disturbances. These symptoms can in turn affect the patients' functional status, general health, and overall quality of life. The impact on individual patients is further modified by psychological and socioeconomic variables [33]. In our study the level of LVEF immediately after the index MI had a statistically significant impact on later HRQoL and in accordance with the usual interpretation of the KCCQ the clinical importance was moderate to large [24]. However, the observed absolute change in *R*-square was small indicating that the amount of variation in HRQoL scores explained by LVEF was moderate. A moderate amount of variation in HRQoL score explained by a single clinical variable can be expected if Wilson and Cleary's model is valid, as other interrelated variables intervene between pathophysiology of the heart and quality of life [33].

Three previous studies have observed an association between LVEF and HRQoL in patients with CAD [8-10]. However, two of these studies reported on patients not comparable to those in the present study. One reported on patients admitted to hospital with acute chest pain, thus including patients with acute MI, unstable angina, and chest pain of other reasons [8]. The second study presented results from patients enrolled in a clinical trial of thrombolysis, thus representing a highly selected group of MI patients [9]. In the third study Ecochard et al. showed that LVEF < 46% measured by ventricular angiography within a month after the index MI was associated with reduced function on the Physical mobility dimension of the Nottingham Health Profile (NHP) assessed at one year [10]. However, they did not observe any association between LVEF and the five other dimensions of the NHP (Energy level, Sleep, Pain, Emotional reactions, and Social isolation). By contrast, we found a relationship between LVEF and composite HRQoL scales covering physical, emotional, and social aspects of the HRQoL concept. Furthermore, we observed falling HRQoL scale scores with falling LVEF for the KCCQ clinical summary score and the EQ-VAS. Ecochard et al. did not find a similar fall by use of NHP, neither did we by use of the EQ-5D Index [10]. The dissimilarities might be due to the fact that both NHP and EQ-5D Index are generic instruments and thus supposed to be less sensitive to smaller changes in health status and disease severity than the disease-specific KCCQ [34]. Another reason why reduced LVEF was not independently associated with lower EQ-5D Index score in our study was the reduced statistical power due to low number of respondents with LVEF < 40%.

Studies that did not find an association between LVEF and later HRQoL also varied with regard to inclusion criteria and most of them reported on selective groups of patients and not on unselected acute MI patients as we did [12-16]. One reported on patients 65 years of age or older with CAD [12], two on clinical trials of post MI patients with reduced LVEF [13,14], and one on patients with their first MI receiving thrombolysis [15]. McBurney et al. did not detect any difference in HRQoL between patients with LVEF < 40% and patients with normal and sub-normal LVEF in a patient sample comparable to ours [16]. However, they used a generic questionnaire, the short form 12 (SF-12) which probably is less sensitive to changes in health status and disease severity [34].

In our study on MI patients, age ≥ 70 years was associated with lower KCCQ clinical summary score. This contrasts with previous results on heart failure patients in which increased age independently correlated with higher KCCQ Quality of life scale score [35]. One possible explanation for this discrepancy is that the KCCQ clinical summery score, which we used as an HRQoL measure in our study, is an overall scale including, in addition to the KCCQ Quality of life scale, scales on symptoms, physical limitations, and social limitations. Another possible explanation is that in our sample of patients with previous MI, increasing age is associated with more advanced CAD and age might act as a surrogate for disease severity [36]. This might not be the case in a sample of patients with heart failure as the underlying cause in younger patients with heart failure more commonly is dilated cardiomyopathy or other cardiomyopathies rather than CAD [35]. We did not observe an association of age with EQ-5D Index scores even though EQ-5D Index US norms report a small decrease in scores with increasing age [32].

We identified sex as an independent determinant of EQ-5D Index score, but not of KCCQ clinical summary score. A difference in score between men and women who are otherwise comparable might be less likely when applying a disease specific instrument than a generic instrument which has a broader perspective. Thus, the EQ-5D Index US norms have reported a small difference between men and women with poorer score in women [32]. Our observations are in agreement with these observations.

Our study showed that the presence of COPD was associated with reduced HRQoL after MI. COPD and CAD share some causal risk factors, and might to some extent present with similar symptoms, for example dyspnoea. Therefore, it is not surprising that patients with COPD who suffer an MI are at increased risk of impaired HRQoL. Similarly, medication for angina pectoris was associated with reduced EQ-5D Index score, and presumably, the reason for this is the fact that such medication reflects enhanced disease severity. In our study the presence of peripheral vascular disease was associated with higher EQ-5D Index score. However, this finding is based on only 11 patients with peripheral vascular disease, and therefore should be interpreted with caution.

Due to the rather long time from the MI to measuring of HRQoL in our study, the association between LVEF and HRQoL could have been distorted. Although the patients reported on intervening major cardiac events, such as new MI or coronary revascularization procedures, other major life events, worsening or improvement of illness not reported in our study, might have had an effect on current HRQoL. We addressed this issue by entering the time between the index MI and HRQoL assessment as a variable in the multivariable analyses. This factor was not, however, independently associated with HRQoL.

In the context of study limitations the representativeness of the sample should be discussed. The eligible patients were representative of survivors of an unselected MI population. Three quarters of the patients responded to the survey, and LVEF was measured in two thirds of the respondents, thus we completed our analysis on approximately half of the eligible patients. This might have skewed the representativeness. However, results from a sub-group analysis of patients in one hospital in which all MI patients had their LVEF measured, were largely in line with the overall results, indicating that the main results pertains to unselected MI patients. Another limitation is the uncertainty of whether the clinicians categorizing the indications for drugs prescribed at discharge did assign the most important indication for each drug, as the protocol instructed. Cardiovascular drugs may have more than one indication for use, and we did not assess the reliability and validity of this classification. However, the classification was undertaken by experienced physicians, most of them cardiologists ensuring the best possible assessment.

## Conclusion

LVEF measured during hospitalization for acute MI is an independent determinant for later HRQoL also after taking sociodemographic and clinical variables into account. The magnitude of the difference in HRQoL score between patients with normal, intermediate, and reduced LVEF was of clinical importance. As expected, in accordance with theoretical models, only a moderate amount of the observed variation in HRQoL score was explained by the level of LVEF.

Restoration of myocardial function after an MI is, in addition to being important for live expectancy, also crucial for long-term daily functioning after the event.

## Abbreviations

CAD: coronary artery disease; COPD: chronic obstructive pulmonary disease; EQ-VAS: EuroQol Visual Analogue Scale; HRQoL: health-related quality of life; KCCQ: Kansas City Cardiomyopathy Questionnaire; LVEF: left ventricular ejection fraction; MI: myocardial infarction; MUGA: multiple gated-acquisition radionuclide ventriculography; NHP: Nottingham Health Profile.

## Competing interests

The authors declare that they have no competing interests. The work was performed at the Norwegian Knowledge Centre for the Health Services and at the Department of Pharmacotherapeutics, University of Oslo, Oslo, Norway. The project had no external funding or grants.

## Authors' contributions

KIP: Had the idea to the study, organized the data collection and data preparation, performed statistical analysis, drafted the first version of the manuscript, revised the manuscript and approved the final version. EK: Participated in data collection and data preparation, participated in discussion of study findings, revision of the manuscript and approved the final version. AR: Participated in discussion of study findings, revision of the manuscript and approved the final version.

KS: Participated in study design, statistical analysis, manuscript preparation and revision, and approved the final version. AR: Participated in data collection, discussion of study findings, manuscript preparation and revision, and approved the final version.

## Pre-publication history

The pre-publication history for this paper can be accessed here:


